# Proximity-Induced Superconductivity in Atomically Precise Nanographene on Ag/Nb(110)

**DOI:** 10.1021/acsmaterialslett.2c00955

**Published:** 2023-03-08

**Authors:** Jung-Ching Liu, Rémy Pawlak, Xing Wang, Hongyan Chen, Philipp D’Astolfo, Carl Drechsel, Ping Zhou, Robert Häner, Silvio Decurtins, Ulrich Aschauer, Shi-Xia Liu, Wulf Wulfhekel, Ernst Meyer

**Affiliations:** † Department of Physics, University of Basel, Klingelbergstrasse 82, 4056 Basel, Switzerland; ‡ Department of Chemistry, Biochemistry and Pharmaceutical Sciences, University of Bern, Freiestrasse 3, Bern 3012, Switzerland; § Physikalisches Institut, Karlsruhe Institute of Technology, Wolfgang-Gaede-Strasse 1, Karlsruhe 76131, Germany

## Abstract

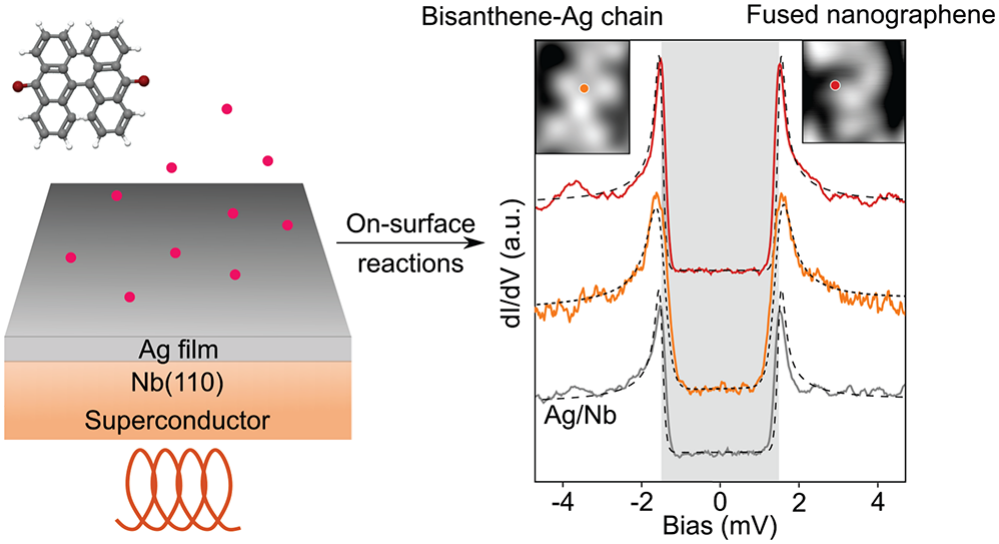

Obtaining a robust superconducting state in atomically precise nanographene (NG) structures by proximity to a superconductor could foster the discovery of topological superconductivity in graphene. On-surface synthesis of such NGs has been achieved on noble metals and metal oxides; however, it is still absent on superconductors. Here, we present a synthetic method to induce superconductivity of polymeric chains and NGs adsorbed on the superconducting Nb(110) substrate covered by thin Ag films. Using atomic force microscopy at low temperature, we characterize the chemical structure of each subproduct formed on the superconducting Ag layer. Scanning tunneling spectroscopy further allows us to elucidate the electronic properties of these nanostructures, which consistently show a superconducting gap.

Topological superconductors are currently of particular interest in condensed matter physics due to their potential as building blocks for topological quantum computation.^1,2^ Topological superconductivity (TS) is elusive in nature, but it can be engineered in hybrid heterostructures by coupling an electron gas with spin-momentum locking to a conventional superconductor.^3−7^ As previously demonstrated in ferromagnetic atomic chains^8−10^ or islands^11^ proximitized with an *s*-wave superconductor, fingerprints of TS are Majorana quasiparticle excitations, so-called Majorana modes. Majorana modes can be identified by scanning tunneling spectroscopy (STS) located at system boundaries, which also mark the system topology.^12^

With bottom-up synthesis through on-surface reactions, atomically precise nanographenes (NGs) and graphene nanoribbons (GNRs)^13,14^ can host Dirac Fermions, topological electronic properties,^15−17^ magnetic edge states,^14,18^ and coupled spins.^19,20^ Despite considerable efforts to engineer their structures and electronic properties on surfaces, observing the interaction of a superconducting state with graphene local magnetism is scarce in literature.^21^ Interestingly, this interaction can lead to further application from strong spin–orbit coupled materials to novel graphene-based topological superconductors,^22^ which opens a new era for implementing Majorana-based *qubits* in topological quantum computation.

Since the synthesis of atomically precise GNRs on Au(111),^13^ the bottom-up fabrication of NGs has become popular for the synthesis of complex graphene structures^23,24^ having well-defined edges^14^ and incorporating topological defects^25,26^ or heteroatomic dopants down to the single atom limit.^27^ Such on-surface reactions require the sublimation of predefined halogen-substituted organic precursors in ultrahigh vacuum (UHV) onto crystalline substrates, which is followed by a thermally triggered surface-assisted polymerization. The crucial part of the polymerization reaction is to initiate an Ullmann-type coupling (dehalogenation), leading to the formation of single C–C bonds between monomers and subsequent cyclizations through cyclodehydrogenation processes. However, these reactions are so far restricted to noble metals (Au, Ag, or Cu)^13,28^ or metal oxides (TiO_2_),^29^ where surface diffusion of molecules, dehalogenation, and cyclodehydrogenation processes are possible by thermal treatment. In contrast, most conventional superconductors are unable to host these reactions due to their low melting points (Pb, In) or high reactivity (Nb, Re). Extending the on-surface chemistry toolbox to superconducting surfaces thus represents a crucial step in the study of the subtle interplay between carbon magnetism and superconductivity.

To date, proximity-induced superconductivity in epitaxial graphene has been achieved by top superconducting electrodes,^30^ by proximity to coadsorbed Pb islands,^21^ or by synthesizing graphene on a superconducting substrate such as Re(0001).^31^ Although the last approach offers the cleanest alternative, as it prevents the contamination of graphene during the fabrication process, the strong hybridization of graphene π bands with 5d Re orbitals was found to prevent Dirac-like electronic dispersion close to the Fermi level.^31^ Later, this issue was circumvented by intercalating a Au buffer layer between graphene and the superconductor,^32^ which restores the intrinsic electronic properties similar to the ones encountered on bulk Au. In our context, such normal metal-superconductor heterostructures are interesting not only for the ability to preserve graphene electronic properties, but also for the capability of hosting surface-assisted reactions.

Inspired by the seminal work of Tomanic et al.,^33^ this work targets Nb(110) substrates covered with thin Ag buffer layers grown in UHV as a reliable superconducting platform.^33^ Using scanning tunneling microscopy (STM) and atomic force microscopy (AFM) at 4.7 K, we investigate Ullmann polymerization of 10,10′-dibromo-9,9′-bianthracene (DBBA) precursors. We characterize the synthesized nanostructures by AFM with CO-terminated tips and confirm the proximity-induced superconductivity. We believe our results open a new route toward the study of topological superconductivity in atomically precise NGs.

Our aim is to reproduce Ullmann polymerization of DBBA precursors (compound **1** in Figure 1a), which leads to seven-carbon-wide armchair GNRs (7-AGNRs) on Ag(111),^28,34^ on the superconducting Ag/Nb(110) substrate. To achieve this, we first investigate by STM the growth of Ag thin films on the oxide-reconstructed Nb(110)^35^ with thicknesses ranging from 0.2 to 5 monolayers (ML) (see Methods and Supporting Information, Figures S2 and S3). Precursor **1** is then sublimed onto the Ag/Nb(110) substrate and kept at room temperature, resulting in 2D self-assembled domains located mostly at step edges and defects (Figure 1b). Upon annealing to *T*_1_ = 75 °C, dehalogenation of **1** is initiated, forming bundles of chains (Figure 1c). Increasing the sample temperature to *T*_2_ = 300 °C then results in single polymeric chains (Figure 1d), while for *T*_3_ = 390 °C, molecular structures appear more curved and fused. The close-up STM image of each subproduct is shown in the insets of Figures 1b–e. We found the formation of these subproducts are independent of the Ag thickness explored so far.

**Figure 1:**
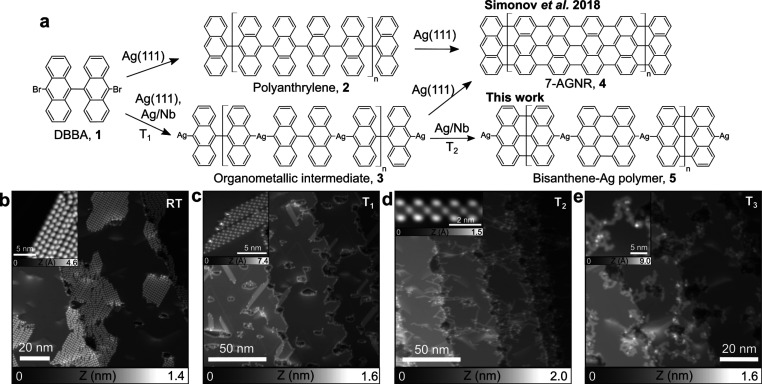
Bottom-up synthesis of NGs on the superconducting Ag/Nb(110) substrate. (a) Hierarchical Ullmann polymerization leading to bisanthene-Ag chains as compared to literature. (b–e) Series of STM images showing the evolution of surface morphology as a function of substrate temperature. (b) Sublimating DBBA molecules (**1**) on Ag/Nb(110) leads to extended two-dimensional self-assemblies (*I*_T_ = 1 pA, *V* = 1.8 V. Inset: *I*_T_ = 1 pA, *V* = −1.5 V). (c) Upon annealing to *T*_1_ = 75 °C, molecular domains evolve to one-dimensional stacks of compounds **3** (*I*_T_ = 1 pA, *V* = −1.5 V. Inset: *I*_T_ = 1 pA, *V* = 900 mV). (d) Annealing to *T*_2_ = 300 °C leads to bisanthene-Ag chains **5** (*I*_T_ = 1 pA, *V* = 1.8 V. Inset: *I*_T_= 1 pA, *V* = 900 mV). (e) The final thermal treatment to *T*_3_ = 390 °C results in small NG domains (*I*_T_ = 1 pA, *V* = 1.9 V. Inset: *I*_T_ = 1 pA, *V* = 2 V).

To better understand the reaction steps, we elucidate the chemical structure of each subproduct using AFM imaging with CO-functionalized tips.^36^Figure 2a and b displays AFM images of zigzag and armchair chains respectively (see Figure S4b and c for corresponding STM images). Both chain configurations are found as intermediates toward 7-AGNRs on noble metal surfaces.^13,28^ In our study, we assign both buckled structures to the formation of organometallic (OM) intermediates **3**, which are composed of bianthracene radicals and Ag surface adatoms. Due to the steric hindrance between anthracene moieties, only the topmost phenyl rings can be resolved by AFM (bold lines in the superimposed Kékulé structures of Figures 2a and b). Our assignment is different from the previous study on Ag(111) using the same precursor,^28^ in which the zigzag pattern was confirmed as polyanthrylene **2** after successful dehalogenation of **1** and formation of C–C bonds. We attribute the two different buckling patterns of **3** on Ag/Nb(110) to the accommodation of the distorted lattice of thin (2.5 ML) Ag films.

**Figure 2:**
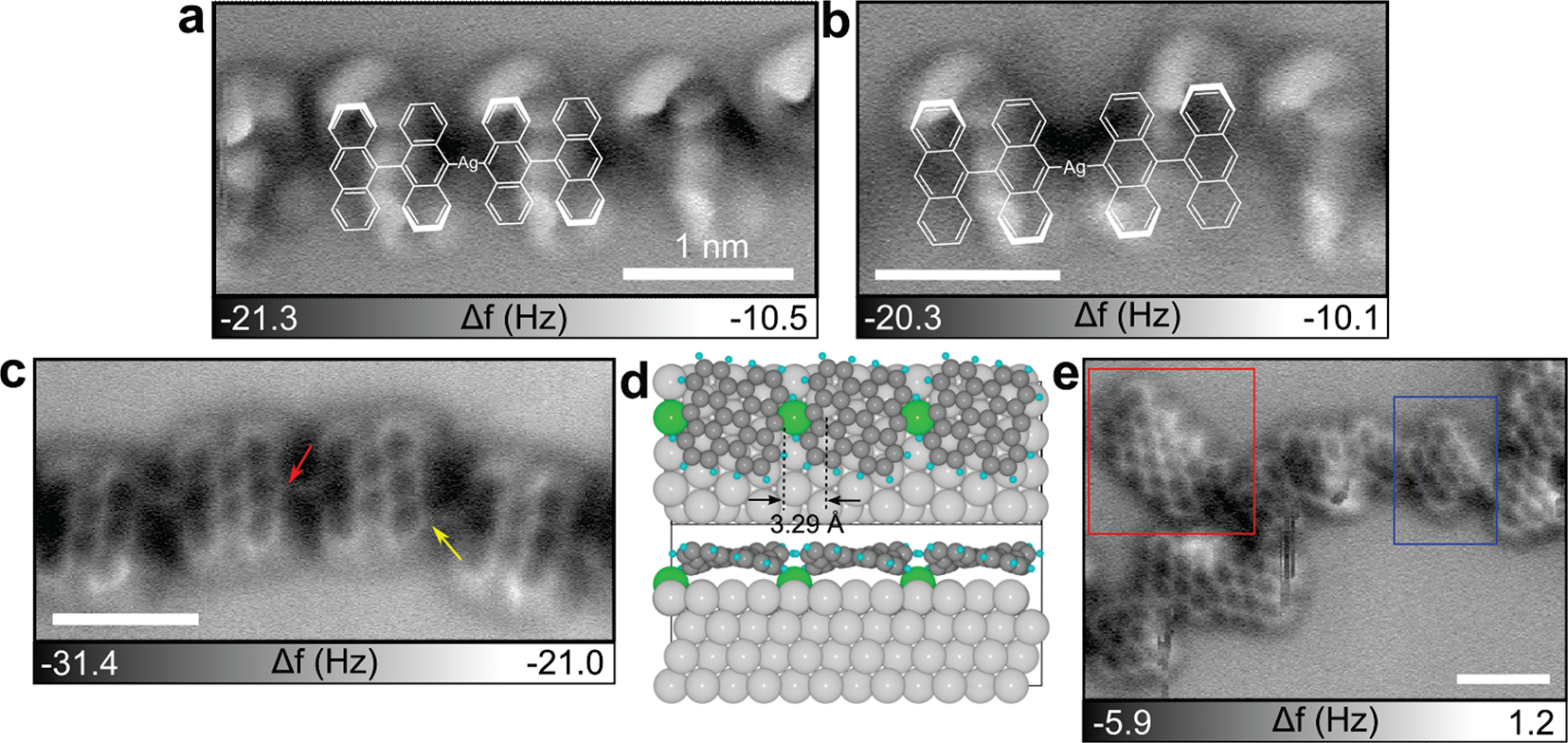
Structural characterization of reaction subproducts. (a) AFM image of OM intermediates **3** showing a zigzag pattern. (b) AFM image of the OM intermediates **3** that have an armchair pattern fingerprint in AFM. Bold bonds in the corresponding Kékulé structures of a and b represent the most protruding part of the chain. Two buckling patterns of **3** might result from the Ag lattice-modulated effect. (c) AFM image of a bisanthene-Ag chain **5** where each monomer is linked by a C–Ag–C bond. (d) Relaxed structure of the OM intermediate on Ag(111) optimized by DFT. The DFT simulation result shows a distance of 3.29 Å between bisanthene moieties. (e) AFM image of the irregularly fused bisanthenes (blue) leading to small 7-GNR segments (red) as well as irregular structures. All scale bars are 1 nm.

To enforce cyclodehydrogenation toward GNRs, we further annealed the sample to *T*_2_ = 300 °C. Exemplary STM/AFM images of the resulting product are shown in the inset of Figure 1d and Figure 2c. Surprisingly, cyclodehydrogenation only occurs within each bianthracene moiety but not between adjacent bisanthene monomers (Figure 2c). By extracting the distance between adjacent bisanthenes, we find the interlinking bond length about 2.51 ± 0.07 Å, which is too long to be a single C–C bond. Moreover, bright protrusions located at the interlinking position in the STM image (inset of Figure 1d) indicate conducting species conjugated in between. Based on these observations, we assign the synthesized structure of Figure 2c to the bisanthene-Ag polymer **5** even though Ag atoms are not clearly resolved by AFM.^37^ According to the relaxed structure of **5** on Ag(111) optimized by density functional theory (DFT) (Figure 2d), surface Ag atoms are pulled out, yielding a distance of 3.29 Å between middle peripheral carbons of adjacent bisanthene monomers. This distance is still much larger than our measured bond length (2.51 Å), which might be explained by different lattice configurations of thin Ag films and Ag(111). Besides, the shorter bond length may be caused by intrinsic limits of the AFM technique, such as the tilted CO molecule at the tip apex^38^ and slight drift during the slow scan.

With the confirmation of **5**, we stress that intermediate **2** is unlikely to be synthesized at this step of the reaction since breaking a strong C–C bond between anthracene moieties and forming a much weaker OM (C–Ag) bond is not energetically favorable. Furthermore, bisanthenes are interlinked not only from the middle of the bisanthene edge (red arrow in Figure 2c) but also from the peripheral carbons (yellow arrow). This observation allows us to conclude that surface Ag atoms of Ag/Nb(110) are involved in the reaction, causing the Ullmann-type reaction to compete with surface-assisted dehydrogenative coupling (non-Ullmann). The synthesis of **5** from DBBA precursors on Ag/Nb(110) is noteworthy, as it is novel compared to previous studies on Ag(111), suggesting that these two substrates do not share identical catalytic reactivity.^28,34^

Annealing the sample to *T*_3_ = 390 °C promotes a cyclodehydrogenation reaction between neighboring bisanthene monomers, leading to dendritic structures (Figure 1e) instead of straight GNRs. These structures consist of irregularly fused bisanthenes or short GNR segments according to AFM imaging (Figure 2e), which are similar to those obtained using chlorinated precursors on Au(111).^39^ We suggest that the Ag coordination between bisanthene monomers prevents the conventional Ullmann reaction (Figure 1a) and promotes alternative cyclodehydrogenation processes. Note that we are aware that kinetic factors such as annealing temperature of the substrate^28^ or heating rate^34^ are key parameters that can influence the reaction toward extended GNRs. We explored various preparation procedures but no significant improvements of Ullmann reaction subproducts nor the formation of extended GNRs have been observed on top of thin Ag film. However, on thicker Ag buffer layers (≥5 monolayer), we observed a Stranski–Krastanov growth mode with the formation of large Ag islands^33^ (Figure S8) on top of which premises of GNR reaction have been obtained with a slow annealing rate (Figure S9). We thus think that the epitaxial deformation in a thin Ag buffer layer containing many surface dislocations (see Figure S2d) leads to a substantial amount of Ag adatoms during annealing for the GNR synthesis. Nevertheless and despite the large involvement of the substrate in the Ullmann reaction, short segments of 7-AGNRs on the thin Ag layer can be occasionally found, as marked by the red rectangle in Figure 2e.

Next, we performed scanning tunneling spectroscopy (STS) on **5** and fused NGs to gain an in-depth understanding of their electronic properties in combination with DFT calculations (see the Methods in the Supporting Information). The superconducting states on **5** were confirmed at 1.3 K (Figure 3a). The overall shape and gap width of the fits using Bardeen-Cooper-Schrieffer (BCS) on **5** are almost identical to those measured on the Ag/Nb(110) substrate (Figure S7), which implies that the superconductivity state given by proximity from the underlying substrate is not influenced much by the molecular adsorbates. In addition, a series of differential conductance (d*I*/d*V*) spectra and d*I*/d*V* mapping were acquired along the central part of **5** (Figure S5b) and across a bisanthene monomer (Figure S5c). Figure 3b shows three representative d*I*/d*V* spectra acquired at positions marked in the inset. Along the chain axis, a shoulder at +0.76 eV is found on bisanthene monomers (orange) and Ag atomic sites (blue) that is absent at the bisanthene armchair edge (green). The latter has a resonance at +0.13 eV attributed to the conduction band (CB) onset. The frontier resonance of the valence band (VB) onset is assigned to −0.60 eV, allowing us to extract an energy gap of about 0.73 eV. In comparison, DFT calculations of the OM structure in gas phase reveal a gap of 1.05 eV, in relative agreement with experimental data. Note that the band gap value extracted from STS measurements is typically reduced by an additional electron screening from the underlying metallic surface with respect to the band gap of the gas-phase polymer obtained by DFT.^40^ Moreover, due to the heavy orbital hybridization with the substrate, a nonzero DOS around Fermi energy as in Figure 3b leads to reasonable observation of superconductivity on **5**.

**Figure 3:**
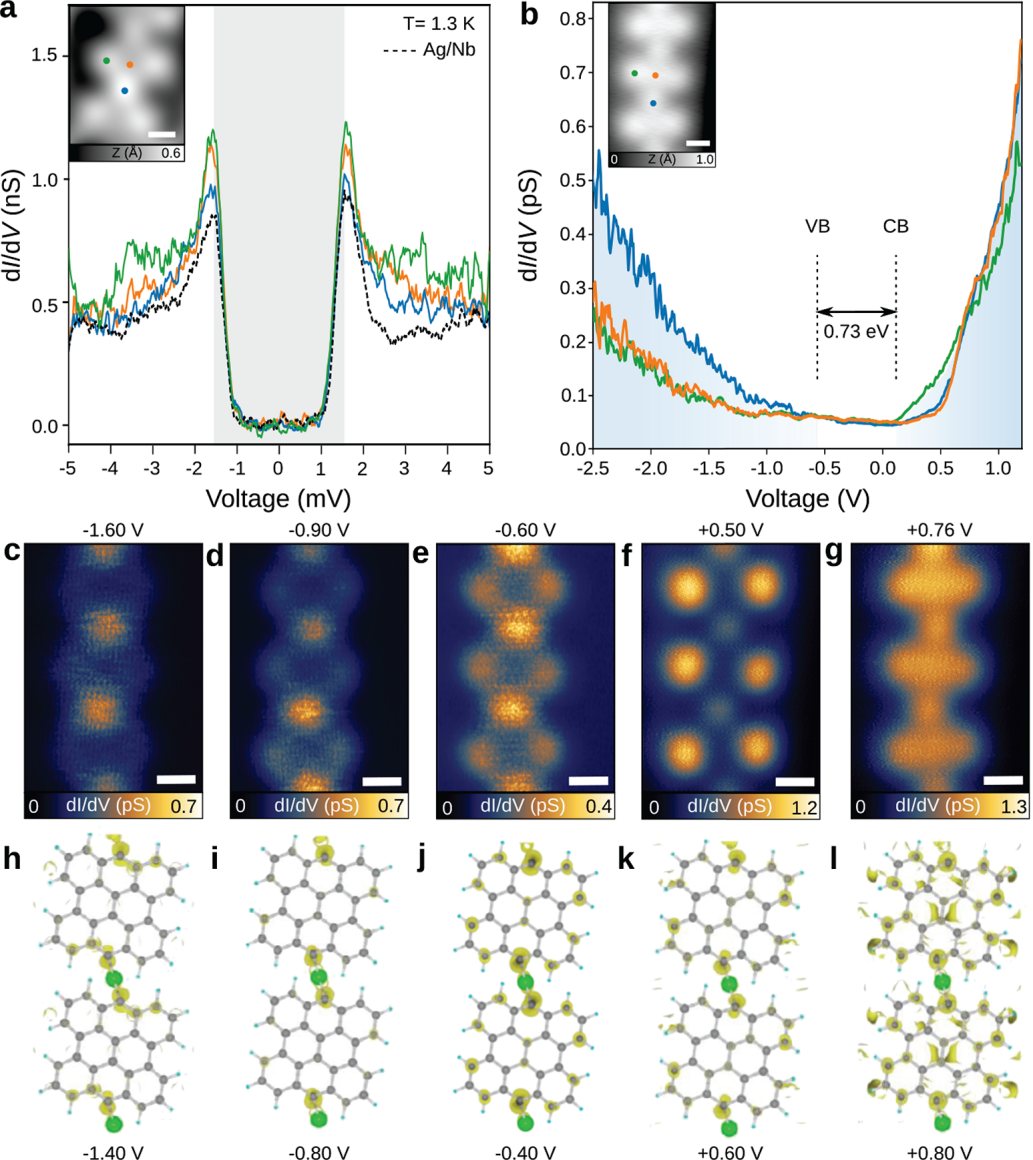
Experimental and simulated electronic properties of bisanthene-Ag chains **5**. (**a**) Proximity-induced superconductivity on **5** at different positions marked in the inset (*I*_t_ = 100 pA, *V* = 10 mV, *A*_mod_ = 50 μV; inset: *I*_t_ = 20 pA, *V* = 760 mV). Dashed line corresponds to representative spectra on pristine Ag/Nb. Shaded area marks the fitted width of the superconducting gap. (b) d*I*/d*V* spectra were measured at three representative positions of the bisanthene chain. Shaded areas refer to the onsets of CB and VB (*I*_t_ = 1 pA, *V* = 900 mV, *A*_mod_ = 20 mV. Inset: *I*_t_ = 1 pA, *V* = 900 mV). (c–g) Series of d*I*/d*V* maps at the indicated bias. Scale bars of all the images are 5 Å. (h–l) Simulated DOS of **5** at different energy levels shows consistency with the experimental results in c–g.

The d*I*/d*V* map acquired at +0.50 eV (Figure 3f) shows an increased density of states (DOS) over bisanthene edges and at Ag atomic sites, while centers of bisanthene units are extinguished. The d*I*/d*V* map above the VB edge (Figure 3c) shows maxima at Ag atoms and lateral termini of the bisanthene moiety, while the d*I*/d*V* map acquired at +0.76 eV (Figure 3g) shows a continuous DOS over the entire chain. Despite the fact that DFT calculations cannot predict correctly the magnitude of the intrinsic band gap of the polymeric chain, we find that the measured DOS of **5** on Ag/Nb(110) has a similar trend to the calculated frontier orbitals of **5** on Ag(111) for the VB and CB band edges (see Figure 3h–l and Figure S6a), validating the character of the frontier orbitals predicted by DFT.

We last discuss the electronic properties of irregularly fused NGs. d*I*/d*V* spectra acquired at different positions share almost identical line shapes (Figure 4a), allowing a gap estimation of 1.58–1.68 eV. We also carry out STS measurements at 560 mK with a metallic tip to investigate the superconductivity within the NG structure. Superconducting gaps at various positions estimated using the Bardeen-Cooper-Schrieffer function considering the thermal broadening effect (Figure S7) have identical widths of Δ = 1.5 meV compared to that on the Ag/Nb(110) substrate (dashed line in Figure 4). Note that we also observe an increase of the SC coherence peak at the edge of the nanographene (red and blue spectra in Figure 4b) as opposed to the center of the NG (green). Such an increase, similar to the one observed for Fe adatom on Nb(110),^41,42^ might suggest the presence of a single pair of YSR states, which energetically overlaps with the coherence peaks due to the limited spectral resolution at the measurement temperature. We thus conclude that the NG structure induces a slight modification of the proximity-induced superconductivity from the Ag/Nb(110) substrate as a result of YSR states likely arising from unpaired electrons at edges of fused bisanthene moieties. This allows us to conclude that the fused NGs on Ag/Nb(110) might contain evidence of carbon magnetism,^20^ which will be explored in future works.

**Figure 4:**
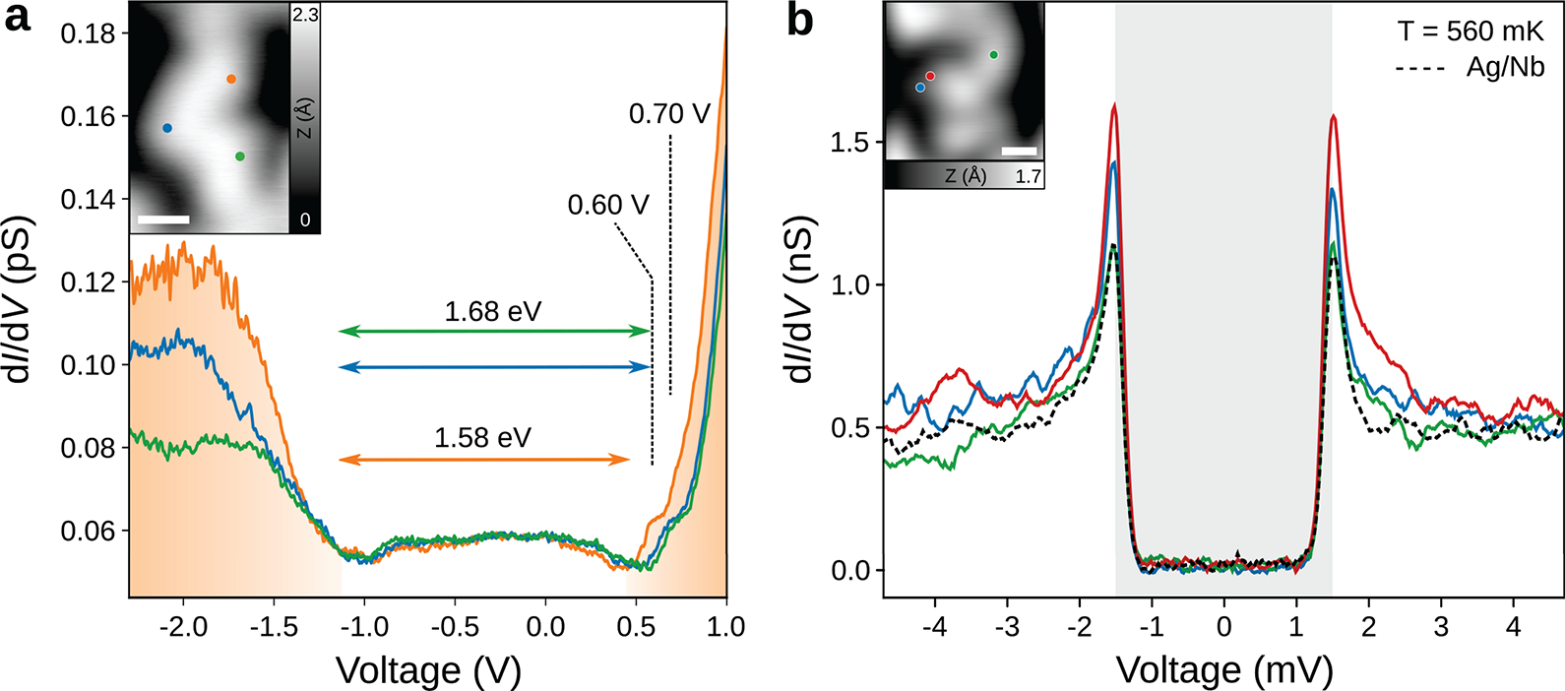
Proximity-induced superconductivity in fused NG. (a) d*I*/d*V* spectra showing VB and CB onsets of a NG segment (*I*_t_ = 1 pA, *V* = 1.8 V, *A*_mod_ = 20 mV). The STM image of the target NG is shown in the inset (*I*_t_ = 1 pA and *V* = 1.8 V). Green, orange, and blue dots in the inset refer to positions of d*I*/d*V* spectra. (b) Superconducting gap measurement at different positions of NG shown in the inset as compared to the pristine Ag/Nb (dashed line) (*I*_t_ = 100 pA, *V* = 10 mV, *A*_mod_ = 50 μV. Inset: *I*_t_ = 50 pA, *V* = 1.5 V). The estimated widths of superconducting gaps (see the Supporting Information, Figure S7) are highlighted by the shaded area, indicating a robust proximity-induced superconductivity on the NG. Scale bars of the insets are 1 nm.

## Conclusion

In conclusion, we fabricated a metal-superconductor heterostructure consisting of a Nb(110) substrate covered by thin Ag films, and found robust proximity-induced superconductivity on the Ag layer.^33^ In contrast to the reactive Nb surface, we demonstrated by low temperature STM/AFM that the Ag buffer layer is compatible with thermal-triggered on-surface reactions, including surface diffusion of molecules, dehalogenation, formation of C–C intramonomer bonds, and cyclodehydrogenation. The presence of surface Ag adatoms from a thin Ag buffer layer, however, changes the reaction pathway compared to pristine Ag(111), leading to unexpected NG structures such as bisanthene-Ag polymeric chains or fused nanographene. We propose to use iodine substituent in future experiments instead of bromine atoms in the precursors to promote polymerization on the surface at lower annealing temperature in order to circumvent the formation of organometallic intermediates.^43^ As compared to the pristine substrate, bisanthene-Ag polymer and edges of fused nanographene show an increase of the SC coherence peaks, which can be attributed to the presence of YSR states from unpaired electrons in these structures.^20^ Our results demonstrate an exciting starting point toward the general exploration of exotic electronic states or carbon magnetism in atomically precise NGs or extended metal–organic frameworks proximitized to a *s*-wave superconductor. This may open new routes toward the emergence of topological superconductivity in carbon-based nanostructures.

## Experimental Methods

### Ag/Nb(110) Preparation

Ag/Nb(110) substrates were prepared under UHV (≈ 10^–10^ mbar) following the protocol described in the previous study.^33^ A Nb(110) substrate purchased from MaTeck GmbH was cleaned by cycles of Ar^+^ sputtering and annealing using a homemade radio frequency (RF) heater. For sample annealing, we carried out five cycles of annealing up to *T* ≥ 1600 °C for 30 s followed by one min of cooling. After careful degassing of the sample, this procedure allowed us to keep the pressure below 5 × 10^–8^ mbar during preparation. Ag films were grown on the Nb(110) surface and kept at room temperature via an e-beam evaporator (EFM3-Focus GmbH). Typical evaporation was about 30 min for a measured flux of about 30–40 nA. After Ag deposition, the sample was annealed with the RF heater to 550 °C for 15 min in order to obtain flat and extended silver monolayers (see Figures S2c–e and S3a). A thicker Ag buffer was obtained by depositing an excessive amount of Ag (with the estimated flux of 15 nA for 1 h 15 min), followed by a substrate annealing. Thick Ag islands with multiple thicknesses due to a Stranski–Krastanov growth were obtained on the thin Ag wetting layer. Temperatures were measured using an infrared pyrometer (Dias Infrared systems GmbH) with an emissivity ξ of 0.12.

### Molecule Deposition

We used DBBA^44^ to perform Ullmann polymerization. DBBA was deposited at 170 °C with the substrate kept at room temperature. The molecule flux was measured by quartz microbalance, which showed 1.38 Å/min at 170 °C. After the deposition, the sample was annealed with the RF heater at different temperatures for 10 min.

### STM/AFM Experiments at 4.7 K

All the samples were characterized at 4.7 K under UHV with the low-temperature STM/AFM provided by Omicron GmbH. The microscope is equipped with a qPlus sensor,^45^ which has a natural oscillation frequency around 23.7 kHz and spring constant 1800 N m^–1^. In order to enhance the AFM resolution, the tip was functionalized with a CO molecule. CO functionalizing was done by depositing CO molecules on the cold surface (≤15 K) and then gently indenting the tip on top of a CO molecule. d*I*/d*V* spectra and maps were recorded with the feedback loop switched off and with the lock-in amplifier using the modulation amplitude indicated in the caption.

### Superconductivity Measurement at Millikelvin Temperature

Superconductivity was investigated at millikelvin temperatures using the dilution refrigerated STM built in Karlsruhe Institute of Technology.^46^ d*I*/d*V* spectra were recorded with the feedback loop switched off and with the lock-in amplifier using the modulation frequency 3.2 kHz and the modulation amplitude of tens of μeV (noted in captions). The width and the position of the superconducting gap were estimated by fitting the spectra with the Bardeen-Cooper-Schrieffer function considering the thermal broadening effect.

### Density Functional Theory (DFT)

DFT calculations were performed using the Quickstep module of CP2K^47^ using the gradient-corrected Perdew–Burke–Ernzerhof (PBE) exchange-correlation functional.^48^ Electron–nuclear interactions were described using Goedecker–Teter–Hutter (GTH) pseudopotentials with 11, 4, and 1 valence electron for Ag, C and H respectively. A molecularly optimized^49^ double-ζ plus polarization (DZVP) basis set was used together with an auxiliary plane-wave basis set with kinetic-energy cutoff of 500 Ry. Reciprocal space was sampled using the Γ point only. Table S1 shows details of the on-surface models used in these calculations. The self-consistent (SCF) calculations were terminated at an energy threshold of 10^–6^ Ha and structures were optimized until forces converged below 4.5 × 10^–4^ Hartree/Bohr.

## Supporting Information Available

Additional STM/STS data on pristine Nb(110) and Ag/Nb(110) substrates; zigzag and armchair intermediate compounds; d*I*/d*V* spectra on the bisanthene-Ag chain; details of DFT calculations of the bisanthene-Ag chain; fits of the SC gap on bisanthene polymer and NG using the BCS function with thermal broadening; on-surface reactions on the thick Ag film (PDF)
