# Supplementary material for: Proximity-Induced Superconductivity in Atomically Precise Nanographene on Ag/Nb(110)
Source: ACS Mater Lett. 2023 Mar 8;5(4):1083–90. doi: 10.1021/acsmaterialslett.2c00955 (PMC10074385; doi:10.1021/acsmaterialslett.2c00955)
Supplement: Supplementary file 1 — tz2c00955_si_001.pdf [file tz2c00955_si_001.pdf]

Supporting Information For "Proximity-Induced Superconductivity in Atomically Precise Nanographene on Ag/Nb(110)"

Jung-Ching Liu,[*,†] Rémy Pawlak,[†] Xing Wang,[‡] Hongyan Chen,[¶] Philipp D'astolfo,[†] Carl Drechsel,[†] Ping Zhou,[‡] Robert Häner,[‡] Silvio Decurtins,[‡] Ulrich Aschauer,[‡] Shi-Xia Liu,[‡] Wulf Wulfhekel,[¶] and Ernst Meyer[*,†]

†*Department of Physics, University of Basel, Klingelbergstrasse 82, 4056 Basel, Switzerland*

‡*Department of Chemistry, Biochemistry and Pharmaceutical Sciences, University of Bern, Freiestrasse 3, 3012 Bern, Switzerland*

¶*Physikalisches Institut, Karlsruhe Institute of Technology, Wolfgang-Gaede-Str. 1, 76131 Karlsruhe, Germany*

E-mail: jungching.liu@unibas.ch; ernst.meyer@unibas.ch

Pristine Nb(110) substrate

The Nb(110) substrate was prepared by Ar^+ sputtering and followed by cycles of heat flash and cooling. As shown in Fig. S1a, bright protrusions and textures of short segments observed in the inset imply that there are still oxides remaining on the surface. The superconductivity of Nb(110) was examined on the sample with less than 1 monolayer (ML) of Ag (red dot in Fig. S1b). Despite of the oxidized surface, the superconducting gap still shows BCS lineshape as in Fig. S1c. The gap was fitted with the thermally broadened

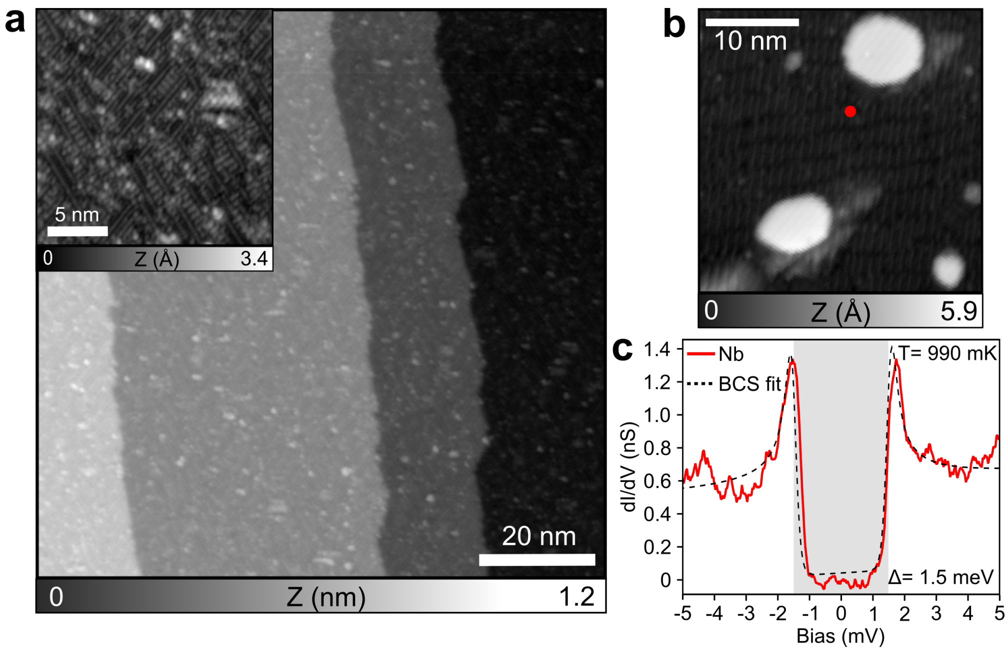

Figure S1: **The STM image and the superconductivity measurement of the pristine Nb(110) substrate. a,** The pristine Nb(110) surface after the cleaning process. The inset is a zoom-in of the surface. The texture of short segments indicates the Nb-O reconstruction (I_t= 1 pA, V= -100 mV). **b,** The Nb(110) surface with Ag coverage of 0.11 ML. **c,** The superconducting gap of Nb measured at the position of the red dot in **b** (I_t= 100 pA, V= 10 mV, A_{mod}= 80 μV). The shaded area marks the width 2Δ, which is also the width of the superconducting gap.

BSC function using the least square method. The fitted Δ= 1.5 meV$\pm 7.3\times10^{-7}$ meV, which is align to the ideal superconducting gap of Nb.

Ag film growth and characterization

To characterize the two-dimensional (2D) growth of Ag films on Nb(110), we keep the Ag evaporation flux and the subsequent annealing process unchanged, and adjust the Ag deposition duration. The Ag coverage is calculated by the software Gwyddion, which shows 21% and 42% of coverage for Fig. S2a and Fig. S2b respectively. The linear relation between the coverage and the deposition time enables intuitive estimation of the Ag film thickness. Thus, the Ag thickness in Figs. S2c, d and e can be deduced as 1.6, 2.5 and 5 ML respectively. On the surface with full coverage of Ag (Figs. S2c-e), we find long triangular

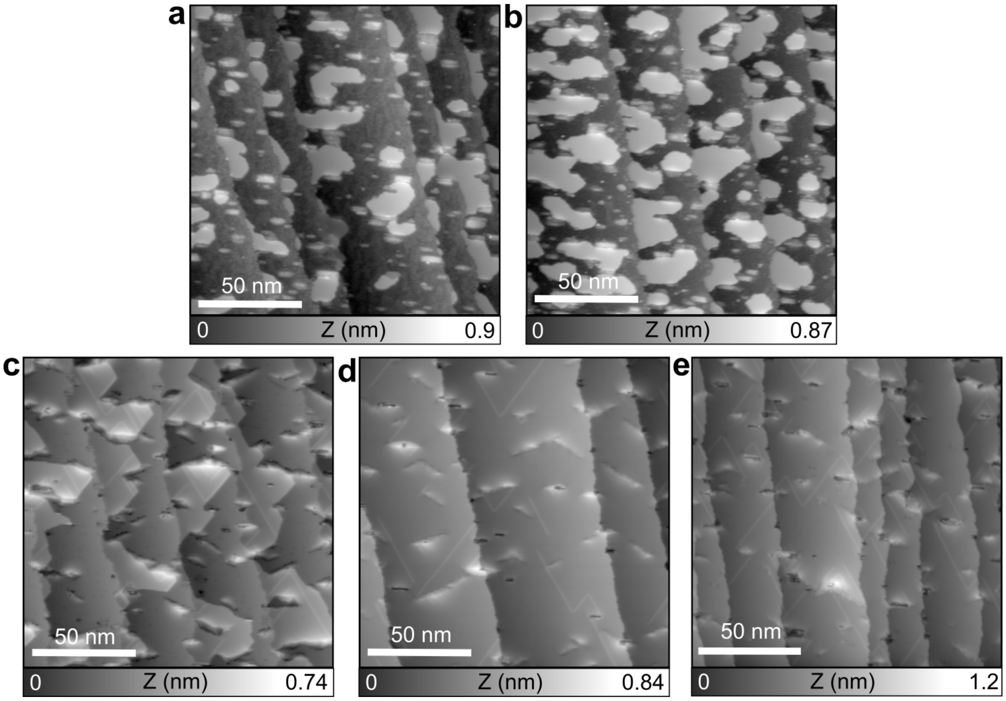

Figure S2: **STM images of different Ag coverages obtained by adjusting the Ag deposition time. a,** 2 minutes 30 seconds (I_t= 1 pA, V= -100 mV), **b,** 5 minutes (I_t= 1 pA, V= -100 mV), **c,** 20 minutes (I_t= 1 pA, V= -100 mV), **d,** 30 minutes (I_t= 1 pA, V= -80 mV), **e,** 1 hour (I_t= 1 pA, V= -80 mV). Terraces are tilted upward in all images to have a better contrast.

lines on terraces, which are considered as the trait of Ag film growth. In addition, defects such as notches at the terrace edge and tiny holes on the terrace are also found on thick Ag.

Since 2.5 ML of Ag provides large and uniform terraces (Fig. S3a), most of the on-surface reactions were performed on the Ag layer with this thickness. The height profile extracted from a clean substrate is plotted in Fig. S3b. The height of a terrace is approximately 0.24 nm, which is consistent with the theoretical height of a Ag(111) atomic layer (0.24 nm). The triangular line is about 35 pm above the surface. The large range $\mathrm{d}I/\mathrm{d}V$ spectra in Fig. S3c exhibits periodically oscillating peaks in the negative bias, and two steps in the positive bias. These signatures could be the result of vertical confinement induced by atomically thin Ag, or the lateral confinement due to limited width of the terrace. Fig. S3d confirms proximity-induced superconductivity on the Ag layer. The superconducting gap is fitted with the same method as Nb, which has Δ= 1.5$\pm$6.3$\times10^{-7}$ meV.

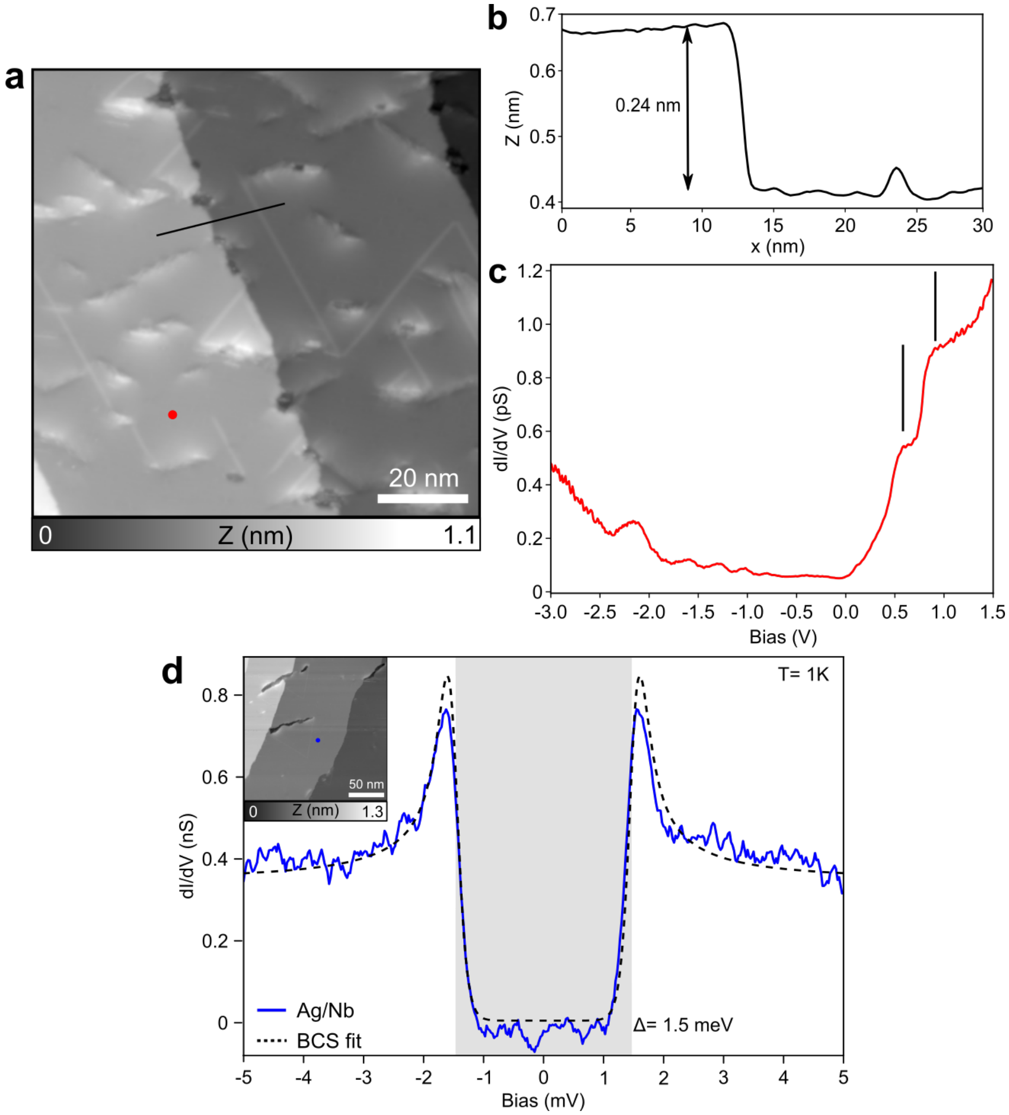

Figure S3: **Characterization of the clean Ag/Nb substrate with 2.5 ML of Ag. a,** The STM image of the clean Ag/Nb surface (I_t= 1 pA, V= -100 mV). **b,** The height profile extracted from the line indicated in **a**. The height of a terrace is roughly 0.24 nm, and the triangular line is about 35 pm above the terrace. **c,** The large scale dI/dV spectrum measured at the position marked in **a** (I_t= 1 pA, V= -1 V, A_{mod}= 20 mV). **d,** The superconducting gap measured on the 2.5 ML Ag at the position indicated in the inset (I_t= 100 pA, V= 10 mV, A_{mod}= 50 μV. Inset: I_t= 100 pA, V= -1 V).

Zigzag and armchair intermediate compounds

After the deposition of DBBA on the Ag/Nb substrate, we found coexisting zigzag and armchair chains (Fig. S4). As discussed in the main text, both of the configurations are organometallic (OM) intermediate compounds towards graphene nanoribbons (GNRs).

The spontaneous conjugation indicates that the energy barrier for forming intermediates is very low, and it can be overcome by the surface-assisted process on this Ag/Nb(110) substrate. Despite of the uniqueness of this certain substrate, OM intermediates are still able to transform to bisanthene-Ag chains by annealing at a much lower temperature estimated to be 150 °C.

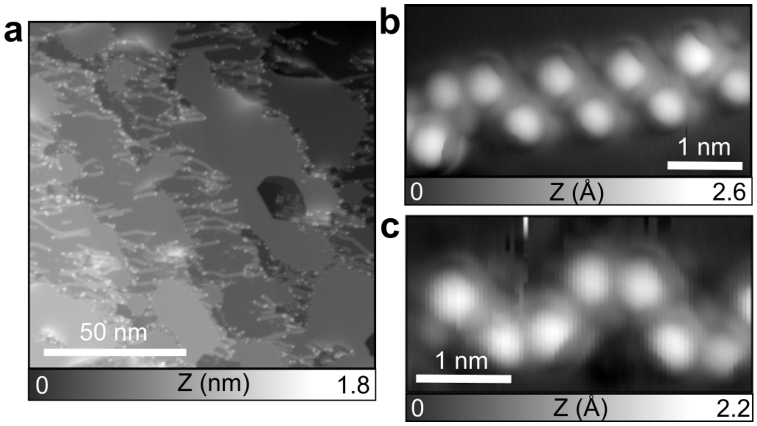

Figure S4: **Structural characterization of zigzag and armchair intermediates. a,** Large scale STM image of the sample where zigzag and armchair intermediates coexist (I_t= 1 pA, V= 1.8 V). **b,** The close-up STM image of a zigzag chain (I_t= 1 pA, V= 150 mV). **c,** The close-up STM image of an armchair chain (I_t= 1 pA, V= 150 mV).

dI/dV spectra on the bisanthene-Ag chain

A series of d$I/$dV spectra were measured along the central axis of a 3-unit bisanthene-Ag chain and across a bisanthene unit as marked in Fig. S5a. For spectra along the central chain axis (Fig. S5b), we find that the onset of the conduction band (CB) is roughly fixed at +0.50 eV while the onset of the valence band (VB) fluctuates between -1.13 eV and -0.60 eV. On the contrary, the measurement across a bisanthene monomer (Fig. S5c) shows an almost identical VB edge while the CB edge oscillates between +0.13 eV and +0.50 eV. As the result, the smallest molecular gap arises at the position of Ag atoms, which has the value of 1.10 eV. In addition, we also find that the resonance state at +0.76 eV is pronounced only along the chain, while it is hardly observed on spectra measured at the bisanthene armchair edge (purple and blue dots in Fig. S5a).

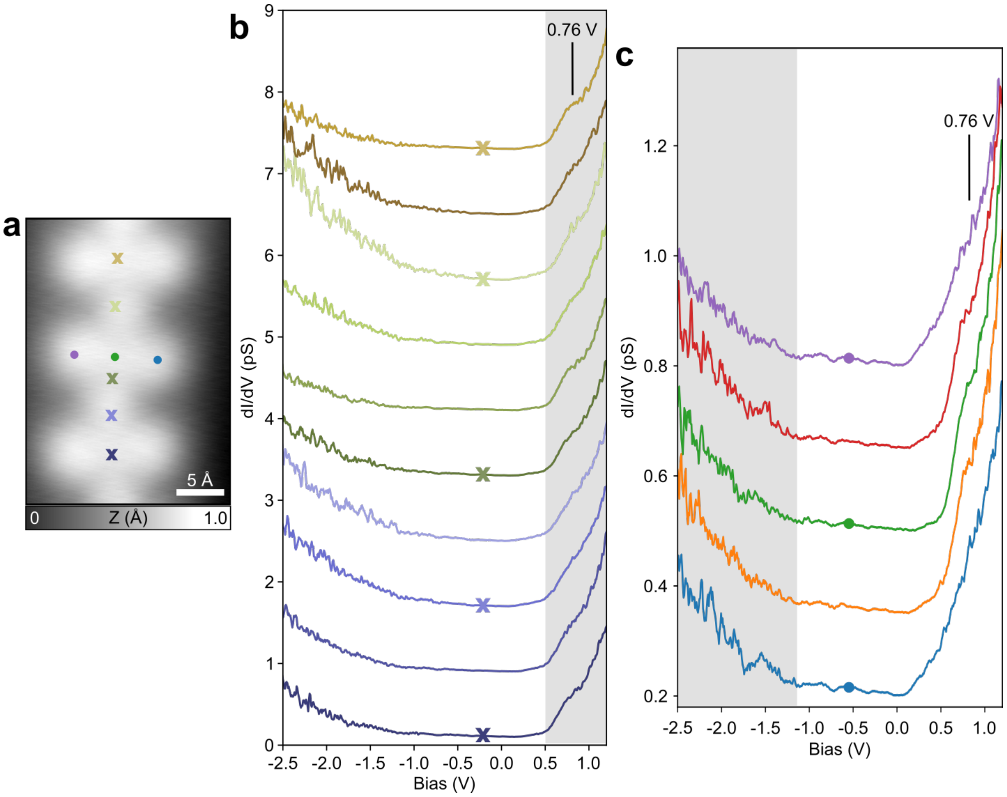

Figure S5: **Complete sets of dI/dV serial spectra measured on the bisanthene-Ag chain.** **a,** The STM image of a 3-unit bisanthene-Ag chain. Crosses and dots in the image mark positions where dI/dV spectra are measured (I_t= 1 pA, V= 900 mV). **b,** Spectra measured at 10 positions along the central axis of the chain. The shaded area marks the conduction band. **c,** Spectra measured at 5 positions across a bisanthene molecule. The shaded area marks the valence band. In **b** and **c**, spectra are vertically shifted to clearly show the lineshape (I_t= 1 pA, V= 900 mV, A_{mod}= 20 mV).

DFT calculations of the bisanthene-Ag chain

Structural calculation. To assist the observation of the novel bisanthene-Ag chain, DFT calculation was performed using Ag(111) as the substrate. Considering different bisanthene-Ag adsorption orientations, the (4×4) orthogonal supercell yields the shortest C-C distance between two adjacent middle peripheral rings (Table S1). The discrepancy in the C-C distance between simulated and measured values is discussed in the main text.

Table S1: **Bisanthene-Ag/Ag(111) supercells used for the on-surface models.** The C-C distance on the (4×4) orthogonal supercell is closest to the experimental value (2.51 Å), thus this structure is used.

Supercells	C-C distance (Å)
(7×5)	3.38
(7×5) with Ag adatom	4.01
(4×5)	4.65
(4×5) with Ag adatom	4.33
(4×4) orthogonal cell	3.29

Density of state (DOS) simulation. Based on the (4×4) orthogonal supercell, the consequent DOS distribution is calculated in Fig. S6a, which shows a similar DOS evolution comparing with acquired dI/dV maps (Figs. 3c-g). Partial density of state (PDOS) is also investigated considering the absence and presence of the Ag(111) substrate (Figs. S6b-c).

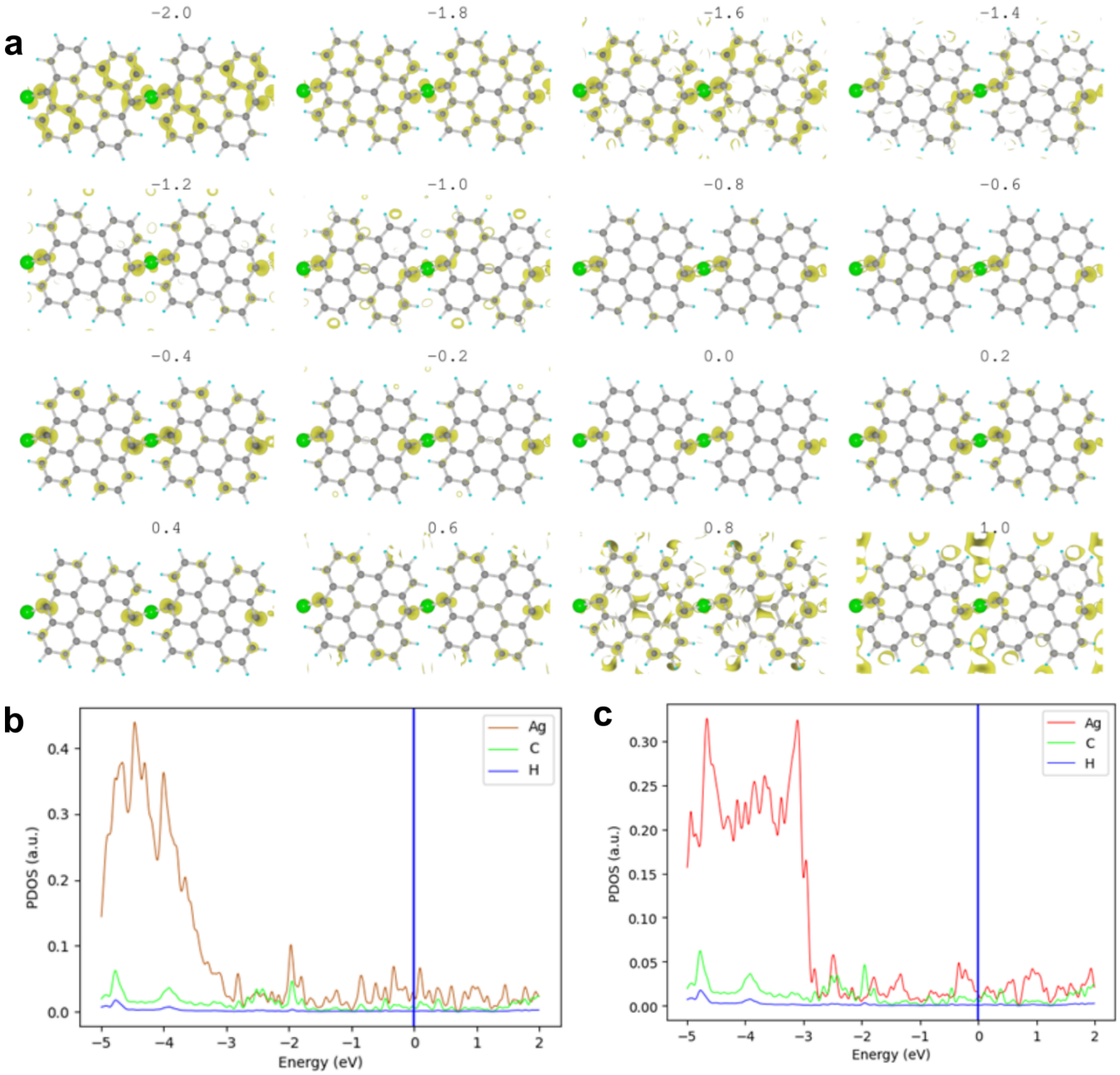

Figure S6: **The simulated DOS of bisanthene-Ag chains at different energy levels. a,** The DOS distribution calculated in the energy range from -2.0 to +1.0 eV, with the interval of 0.2 eV. The simulation results show a similar evolution trend with acquired $\mathrm{d}I/\mathrm{d}V$ maps. **b-c,** PDOS calculated without (**b**) and with (**c**) the Ag(111) substrate.

Superconducting gaps on the bisanthene-Ag chain and NG

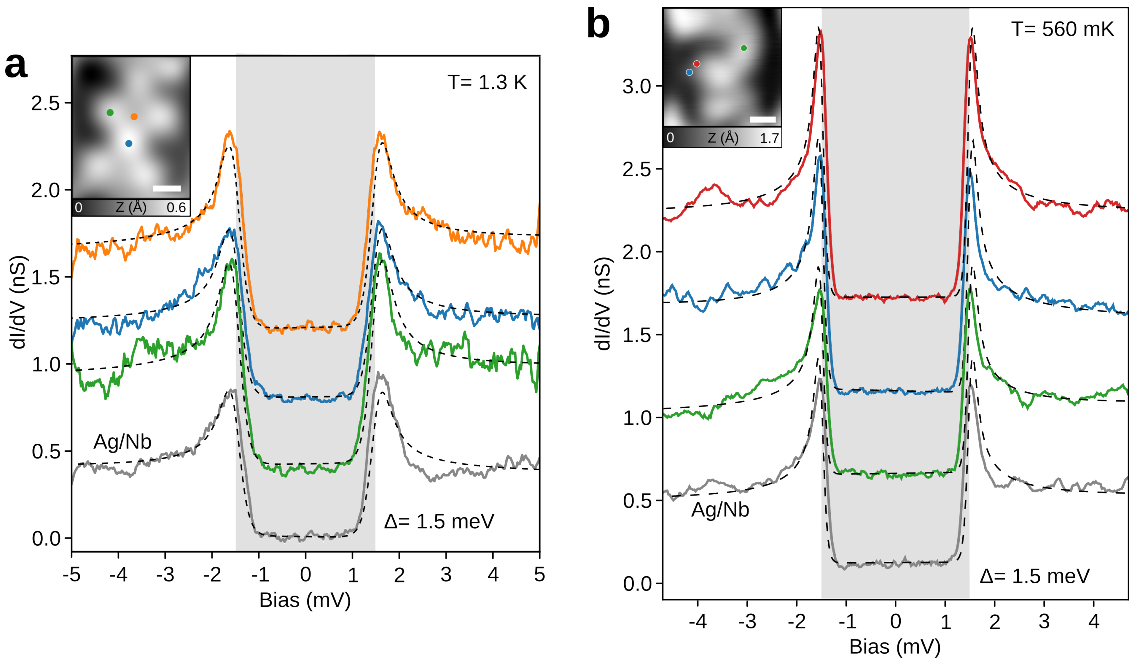

Figure S7: **Fits of the SC gap on bisanthene polymer and NG using BSC function with thermal broadening.** Superconducting gap measurement of the bisanthene-Ag chains (**a**), and fused nanographene (**b**). Dashed lines correspond to the BSC fits considering thermal broadening effect giving a width of $\Delta = 1.5$ meV. The estimated widths of superconducting gaps are highlighted by the shaded area, indicating a robust proximity-induced superconductivity. Curves are vertically shifted for clarity.

On-surface reactions on the thick Ag film

On-surface reactions of DBBA were also performed on thick Ag layer ($\geq$ 5 ML) as comparison with thin Ag layer. We occasionally found stripe structures as shown in Fig. S9 by annealing the sample to 330 °C over 30 minutes. These structures differs from the ones reported in the main manuscript and can be attributed to GNRs. We wish to explore them by AFM imaging in future works.

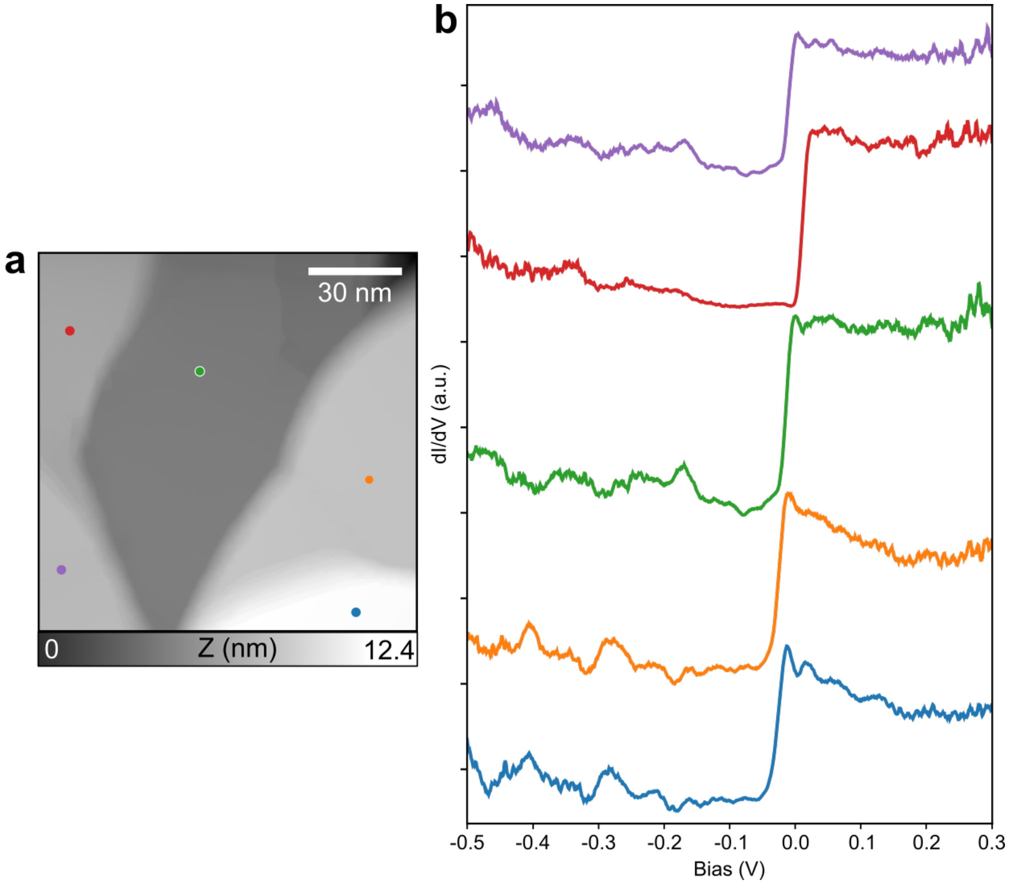

Figure S8: **Characterization of the thick Ag layer grown on Nb(110). a,** STM topographic image of the Ag layer showing a Stranki-Krastanov growth mode (I_t= 100 pA, V= 800 mV). **b,** Surface state is probed by dI/dV at different positions of the surface ($\approx$ -10 mV) below the Fermi level (I_t= 100 pA, V= -500 mV, A_{mod}= 10 mV). As reported by Tomanic *et al.*[1], the surface state is found to become fully superconducting for such coverage. We thus speculate that GNRs synthesized on such Ag films might show similar superconducting gap as reported in the main manuscript.

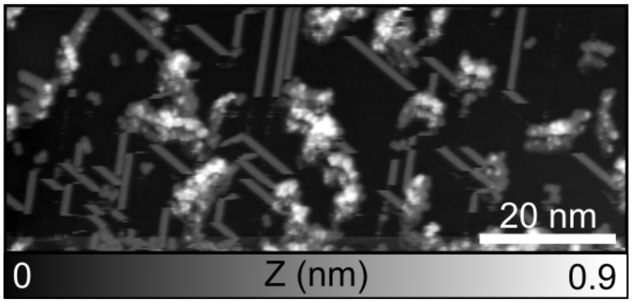

Figure S9: **STM images of the products synthesized on the thick Ag layer.** Stripe-like structures are synthesized on the thick Ag after the slow annealing process that could be attributed to GNRs (I_t= 100 pA, V= 1 V).

References

(1) Tomanic, T.; Schackert, M.; Wulfhekel, W.; Sürgers, C.; Löhneysen, H. v. Two-band superconductivity of bulk and surface states in Ag thin films on Nb. *Phys. Rev. B* **2016**, *94*, 220503.
